# Supplementary material for: Revisiting typing systems for group B Streptococcus prophages: an application in prophage detection and classification in group B Streptococcus isolates from Argentina
Source: Microb Genom. 2024 Oct 17;10(10):001297. doi: 10.1099/mgen.0.001297 (PMC11485964; doi:10.1099/mgen.0.001297)
Supplement: Uncited Supplementary Material 1. [file mgen-10-01297-s001.pdf]

# Detailed Materials and Methods

## M3. Prophage detection and typing.

In the first instance, prophage sequences were detected and preliminarily classified using the previous screening methods for prophage groups [1] and integrase type [2], followed by a manual curation as follows:

1- Prophage groups A-F were established using an *in silico* PCR with ipcress (part of the exonerate package v2.4.0), [3], based on the 12 primer pairs designed by van der Mee-Marquet et al. [1]; a strain was considered to be potentially carrying a prophage when a positive result (sequence of the expected size amplified) was obtained with at least one primer pair.

2- The integrase type was defined based on the method by Crestani et al [2]: a blastx (Blast v2.9.0+) [4] search was executed with genome assemblies as query, against the GBS prophage integrase database; an integrase type (GBS*Int*) for the potential prophage was assigned when there was at least 90% of sequence identity over 95% of the sequence length.

3- Results of the two screening methods were combined corroborating that the detected genes were located on the same contig. The putative prophages were classified into prophage types according to prophage group and integrase type.

4- To verify the presence of the screened prophages, the navigator function of Artemis v17.0.1 [5] was used to manually search for the genes and sequences of interest within the assembled genomes. The prophages with an assigned integrase type were located directly by searching for the *att* sequences corresponding to the integrase type, while those without an assigned integrase type were located by searching for the phage group-determining genes and the environment of these genes was manually explored to delimit the beginning and end of the prophages.

All prophages were annotated with RASTtk v2.0 in the RAST server [6–8], customising the default pipeline to prioritise the annotation of phage proteins.

If a prophage sequence was fragmented across two or more contigs, the prophage was reconstructed as follows:

1- A megablast search was performed to compare each fragmented prophage against non-fragmented prophage sequences. The prophage with the highest identity and coverage (>99% and >80%, respectively) was used as reference for mapping the raw reads of genomes harbouring fragmented prophages.

2- Smalt v0.7.6 [9] was used for mapping the reads against the reference genomes and the analysis and organisation of the reads was performed with SAMtools v1.12 [10].

3- The mapped reads were extracted and used for *de novo* assembly with SPAdes v3.13.1 [11].

4- The newly assembled prophages were annotated with RASTtk and compared with the original contigs to confirm consistent coding sequence content.

A phylogenetic tree was constructed to analyse the relationships between all prophages detected, as follows:

1- The extracted prophage sequences were aligned with MAFFT v7.505 [12], using CIPRES Science Gateway [13], with default parameters.

2- A maximum-likelihood (ML) phylogenetic tree was reconstructed with IQTree v1.6.12 [14], with ModelFinder [15] to determine the best-fit model with 1000 SH-aLRT [16] and 1000 ultrafast-bootstrap [17] replicates.

Prophages that could not be assigned to a prophage group by the *in silico* PCR during the initial screening stage were classified as belonging to the same prophage group as other classified prophages in their phylogenetic cluster.

## M4. Improvement of the prophage typing system

To improve the integrated screening system and to avoid false positive and false negative results with the *in silico* PCR, a new prophage group detection method is here proposed. The method was developed as follows:

1- A database was built with the genes amplified by primers developed by van der Mee-Marquet [1].

a) A blastn search was performed in the prophages to verify that the same results were obtained as by the *in silico* PCR.

b) The 22 prophages detected by van der Mee-Marquet et al. [1] were included as positive control.

2- The database was curated as follows (Table S1 in S2 File ):

a) New genes were selected from the control and Argentinean prophage sequences and added to the database, as representatives for non-typable prophage groups.

b) Genes responsible for false positive results were replaced.

3- A new blastn search was performed against the enriched database, to test the methodology on the 22 prophages detected by van der Mee-Marquet and on those obtained from Argentinean GBS genomes. A positive result was considered when at least one of the genes for the prophage group was detected with a minimum of 75% of identity and coverage.

4- To ensure the new method was effective, the blastn search was repeated using as query the whole genomes of:

a) the 365 GBS from Argentina and

b) a dataset of 250 GBS isolated from humans and animals in 34 countries on 5 continents, of a wide variety of clonal complexes and temporal origins, downloaded from NCBI (https://www.ncbi.nlm.nih.gov/, May 2023) (Table S2 in S2 File).

5- The results were integrated with blastx results for integrase type searches [2].

When prophages with integrase subtypes (n=2) not described by Crestani et al. were found, the criteria described by the authors were followed for their classification: integrase genes with the same insertion site but with aminoacid sequence identity below 90% were classified as the same type but different subtype [2].

## M5. Prophage characterization.

Several steps were followed for the characterization of all GBS prophages found:

1- Prophage sequences were searched for genetic determinants of virulence and antimicrobial resistance with:

a) ABRicate v0.9.9 [18], using VFDB (version 2023-06-27) [19] and ResFinder (version 2023-06-27) [20] databases, respectively, and

b) AMRFinderPlus v3.11.4 (database version 2023-04-17.1) [21].

2- The SEED Viewer v2.0 [7] was used to browse the RASTtk annotations to search for prophage genes potentially beneficial for the host bacteria.

3- Genes coding for integrase, helicase, terminase large subunit, major capsid protein and lysin, were used for the phylogenetic analysis of each prophage module, as described in section M3.

One phage of each prophage type (n=29, see results section) was selected for further characterization:

1- The morphology of the prophages was determined by the recognition of their head-neck-tail modules with VIRFAM [22].

2- CDD [23], CDART [24] and InterPro [25] were used to identify the function of the prophage genes annotated as encoding hypothetical or phage proteins and also to analyse the catalytic domain of the putative integrases and lysins present.

3- To analyse the genetic differences between prophages of the same prophage group but different integrase type and *vice versa*, a comparative analysis of the prophage sequences was performed with clinker v0.027 [26].

To provide a broader context to the prophages from Argentinian GBS, 70 GBS prophages were accessed from NCBI (May 2023) alongside 369 prophages of other 43 streptococcal species identified by Rezaei Javan et al. [27] for a total of 764 prophages (Table S3 in S2 File). All prophages were aligned with MAFFT, and a ML phylogenetic tree was reconstructed using IQTree, as described in section M3. Additionally, the prophage type of GBS prophages from NCBI was determined as described in M3 in this file.

## Supplementary References


1. van der Mee-Marquet N, Diene SM, Barbera L, Courtier-Martinez L, Lafont L, Ouachée A, et al. Analysis of the prophages carried by human infecting isolates provides new insight into the evolution of Group B Streptococcus species. Clin Microbiol Infect. 2018;24: 514–521. doi:10.1016/j.cmi.2017.08.024
2. Crestani C, Forde TL, Zadoks RN. Development and Application of a Prophage Integrase Typing Scheme for Group B Streptococcus. Front Microbiol. 2020;11: 1993. doi:10.3389/fmicb.2020.01993
3. Slater G, Birney E. Automated generation of heuristics for biological sequence comparison. BMC Bioinformatics. 2005;6: 31. doi:10.1186/1471-2105-6-31
4. Camacho C, Coulouris G, Avagyan V, Ma N, Papadopoulos J, Bealer K, et al. BLAST+: architecture and applications. BMC Bioinformatics. 2009;10: 421. doi:10.1186/1471-2105-10-421
5. Carver T, Harris SR, Berriman M, Parkhill J, McQuillan JA. Artemis: an integrated platform for visualization and analysis of high-throughput sequence-based experimental data. Bioinformatics. 2012;28: 464–469. doi:10.1093/bioinformatics/btr703
6. Aziz RK, Bartels D, Best AA, DeJongh M, Disz T, Edwards RA, et al. The RAST Server: Rapid Annotations using Subsystems Technology. BMC Genomics. 2008;9: 75. doi:10.1186/1471-2164-9-75
7. Overbeek R, Olson R, Pusch GD, Olsen GJ, Davis JJ, Disz T, et al. The SEED and the Rapid Annotation of microbial genomes using Subsystems Technology (RAST). Nucleic Acids Res. 2014;42: D206–D214. doi:10.1093/nar/gkt1226
8. Brettin T, Davis JJ, Disz T, Edwards RA, Gerdes S, Olsen GJ, et al. RASTtk: A modular and extensible implementation of the RAST algorithm for building custom annotation pipelines and annotating batches of genomes. Sci Rep. 2015;5: 8365. doi:10.1038/srep08365
9. Ponstingl H. SMALT. Wellcome Trust Sanger Institute, Cambridge, UK; 2010. Available: https://www.sanger.ac.uk/tool/smalt/
10. Danecek P, Bonfield JK, Liddle J, Marshall J, Ohan V, Pollard MO, et al. Twelve years of SAMtools and BCFtools. GigaScience. 2021;10: giab008. doi:10.1093/gigascience/giab008
11. Bankevich A, Nurk S, Antipov D, Gurevich AA, Dvorkin M, Kulikov AS, et al. SPAdes: A New Genome Assembly Algorithm and Its Applications to Single-Cell Sequencing. J Comput Biol. 2012;19: 455–477. doi:10.1089/cmb.2012.0021
12. Katoh K, Standley DM. MAFFT Multiple Sequence Alignment Software Version 7: Improvements in Performance and Usability. Mol Biol Evol. 2013;30: 772–780.

doi:10.1093/molbev/mst010

13. Miller MA, Pfeiffer W, Schwartz T. Creating the CIPRES Science Gateway for inference of large phylogenetic trees. Proceedings of the Gateway Computing Environments Workshop (GCE). New Orleans, LA; 2010. p. pp 1-8.
14. Minh BQ, Schmidt HA, Chernomor O, Schrempf D, Woodhams MD, von Haeseler A, et al. IQ-TREE 2: New Models and Efficient Methods for Phylogenetic Inference in the Genomic Era. Teeling E, editor. Mol Biol Evol. 2020;37: 1530–1534. doi:10.1093/molbev/msaa015
15. Kalyaanamoorthy S, Minh BQ, Wong TKF, Von Haeseler A, Jermiin LS. ModelFinder: fast model selection for accurate phylogenetic estimates. Nat Methods. 2017;14: 587–589. doi:10.1038/nmeth.4285
16. Guindon S, Dufayard J-F, Lefort V, Anisimova M, Hordijk W, Gascuel O. New Algorithms and Methods to Estimate Maximum-Likelihood Phylogenies: Assessing the Performance of PhyML 3.0. Syst Biol. 2010;59: 307–321. doi:10.1093/sysbio/syq010
17. Minh BQ, Nguyen MAT, von Haeseler A. Ultrafast Approximation for Phylogenetic Bootstrap. Mol Biol Evol. 2013;30: 1188–1195. doi:10.1093/molbev/mst024
18. Seemann T. Abricate, Github https://github.com/tseemann/abricate. 2020.
19. Chen L, Zheng D, Liu B, Yang J, Jin Q. VFDB 2016: hierarchical and refined dataset for big data analysis—10 years on. Nucleic Acids Res. 2016;44: D694–D697. doi:10.1093/nar/gkv1239
20. Zankari E, Hasman H, Cosentino S, Vestergaard M, Rasmussen S, Lund O, et al. Identification of acquired antimicrobial resistance genes. J Antimicrob Chemother. 2012;67: 2640–2644. doi:10.1093/jac/dks261
21. Feldgarden M, Brover V, Gonzalez-Escalona N, Frye JG, Haendiges J, Haft DH, et al. AMRFinderPlus and the Reference Gene Catalog facilitate examination of the genomic links among antimicrobial resistance, stress response, and virulence. Sci Rep. 2021;11: 12728. doi:10.1038/s41598-021-91456-0
22. Lopes A, Tavares P, Petit M-A, Guérois R, Zinn-Justin S. Automated classification of tailed bacteriophages according to their neck organization. BMC Genomics. 2014;15: 1027. doi:10.1186/1471-2164-15-1027
23. Wang J, Chitsaz F, Derbyshire MK, Gonzales NR, Gwadz M, Lu S, et al. The conserved domain database in 2023. Nucleic Acids Res. 2023;51: D384–D388. doi:10.1093/nar/gkac1096
24. Geer LY. CDART: Protein Homology by Domain Architecture. Genome Res. 2002;12: 1619–1623. doi:10.1101/gr.278202
25. Paysan-Lafosse T, Blum M, Chuguransky S, Grego T, Pinto BL, Salazar GA, et al.

InterPro in 2022. Nucleic Acids Res. 2023;51: D418–D427. doi:10.1093/nar/gkac993

26. Gilchrist CLM, Chooi Y-H. clinker & clustermap.js: automatic generation of gene cluster comparison figures. Robinson P, editor. Bioinformatics. 2021;37: 2473–2475. doi:10.1093/bioinformatics/btab007
27. Rezaei Javan R, Ramos-Sevillano E, Akter A, Brown J, Brueggemann AB. Prophages and satellite prophages are widespread in Streptococcus and may play a role in pneumococcal pathogenesis. Nat Commun. 2019;10: 4852. doi:10.1038/s41467-019-12825-y

# Supplementary Figures

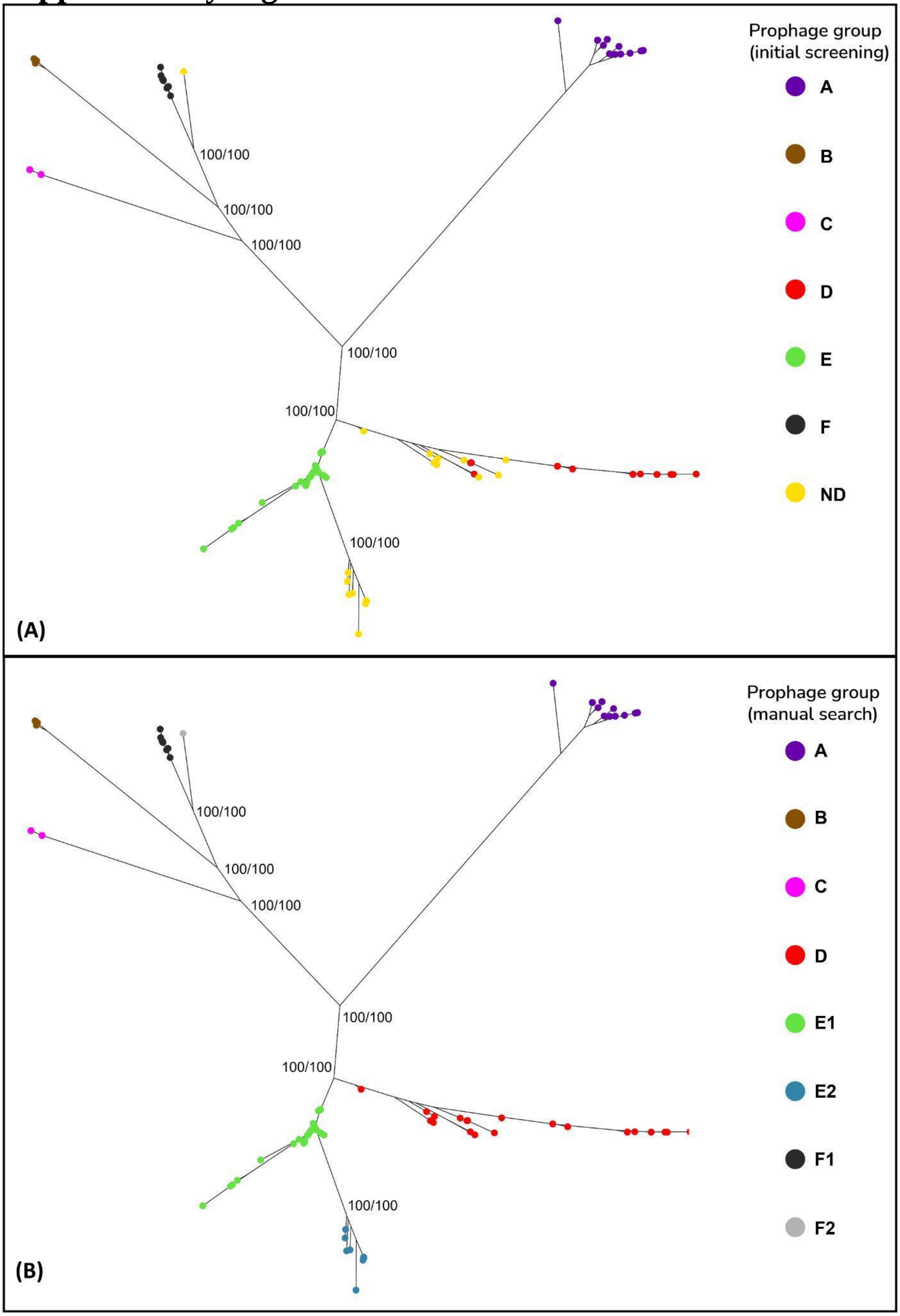


**Fig S1. Phylogeny of the 325 prophages found in 365 GBS genomes from Argentina.** Maximum likelihood phylogenetic tree with nodes coloured by prophage group. Ultrafast-bootstrap values are shown as labels in the main clusters. (A) Prophage groups determined by the original screening by *in silico* PCR; prophages classified as ND (coloured in yellow) were not detected by the PCR so their prophage group was not determined. (B) Prophage groups determined after the manual search of the prophages and their phylogenetic analysis; prophages previously classified as ND were assigned the prophage group corresponding to their cluster. https://microreact.org/project/philogeny-argentinean-gbs-prophages

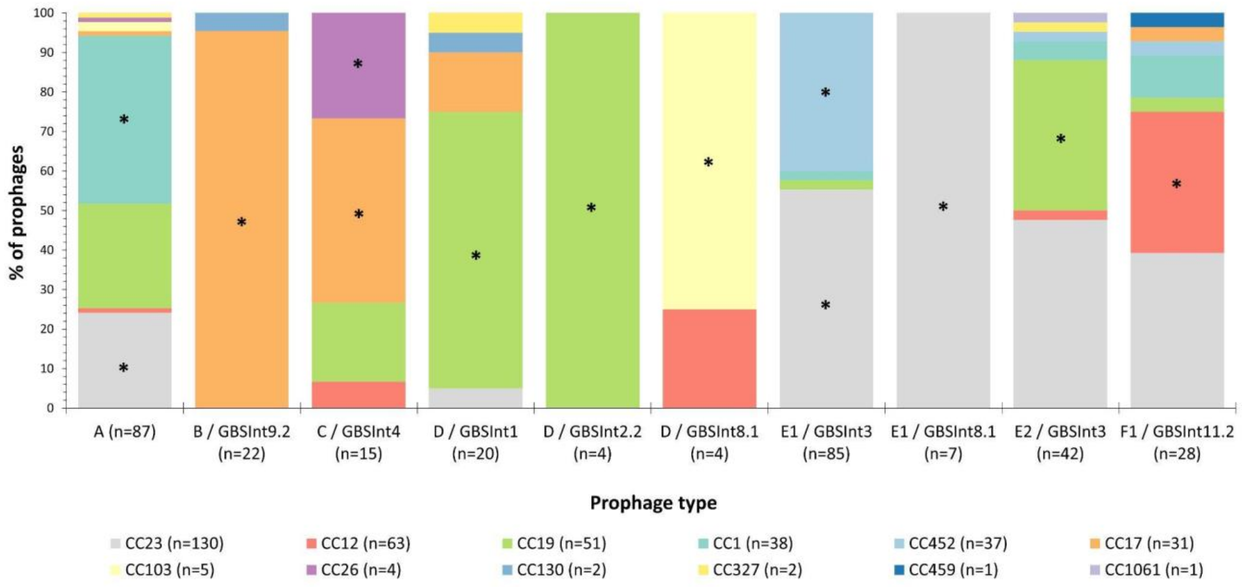


**Fig S2. Prophage distribution in GBS isolates from Argentina according to clonal complex.** Only the prophage types with n > 2 are shown. Significant associations ($p<0.05$) between prophage type and CC are marked with *.

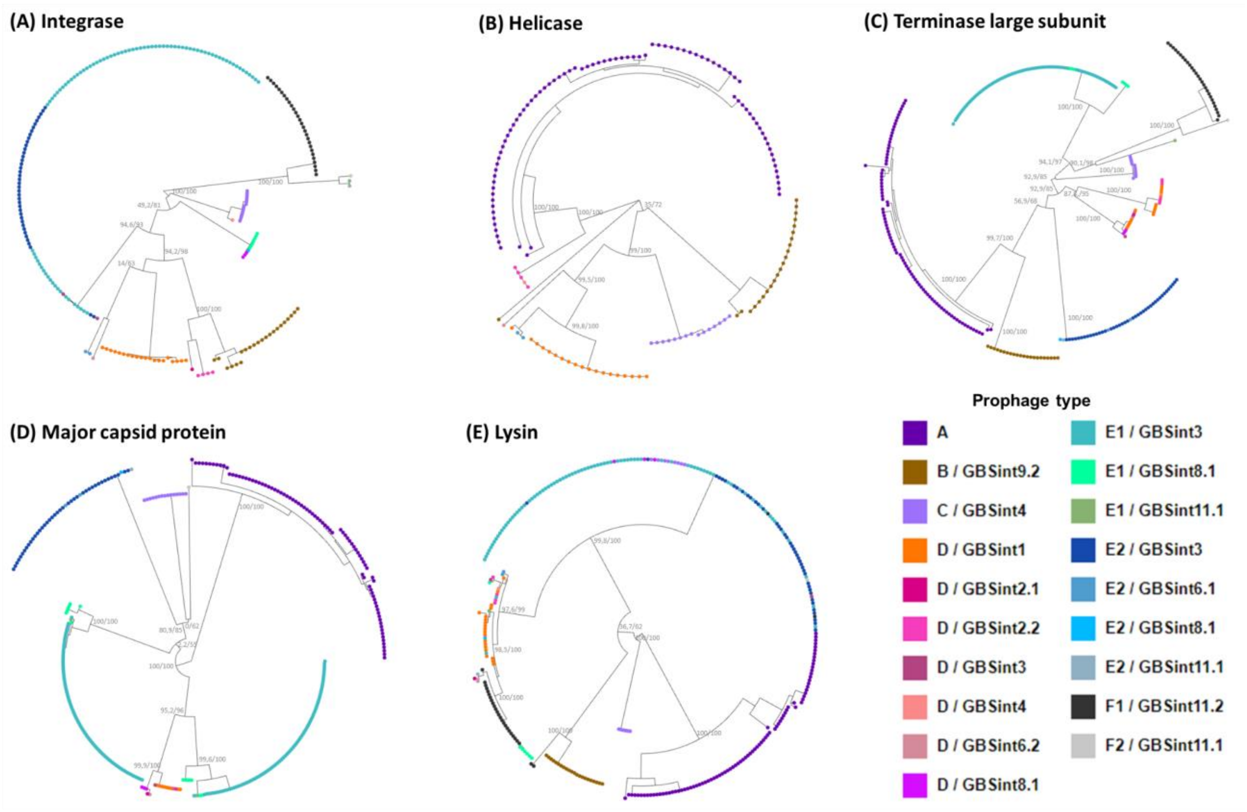


**Fig S3. Phylogeny of selected modular genes of the 325 GBS prophages from Argentina.** Maximum likelihood phylogenetic trees, midpoint rooted, based on the aligned nucleotide sequences of the genes coding for (A) integrase (lysogeny module), (B) helicase (replication module) (C) terminase large subunit (packaging module), (D) major capsid protein (morphogenesis module) and (E) lysin (host lysis module). Tree nodes are coloured by prophage type. Support values (SH-aLRT/ Ultrafast Bootstrap) are shown as labels for selected nodes. https://microreact.org/project/philogeny-of-modular-genes

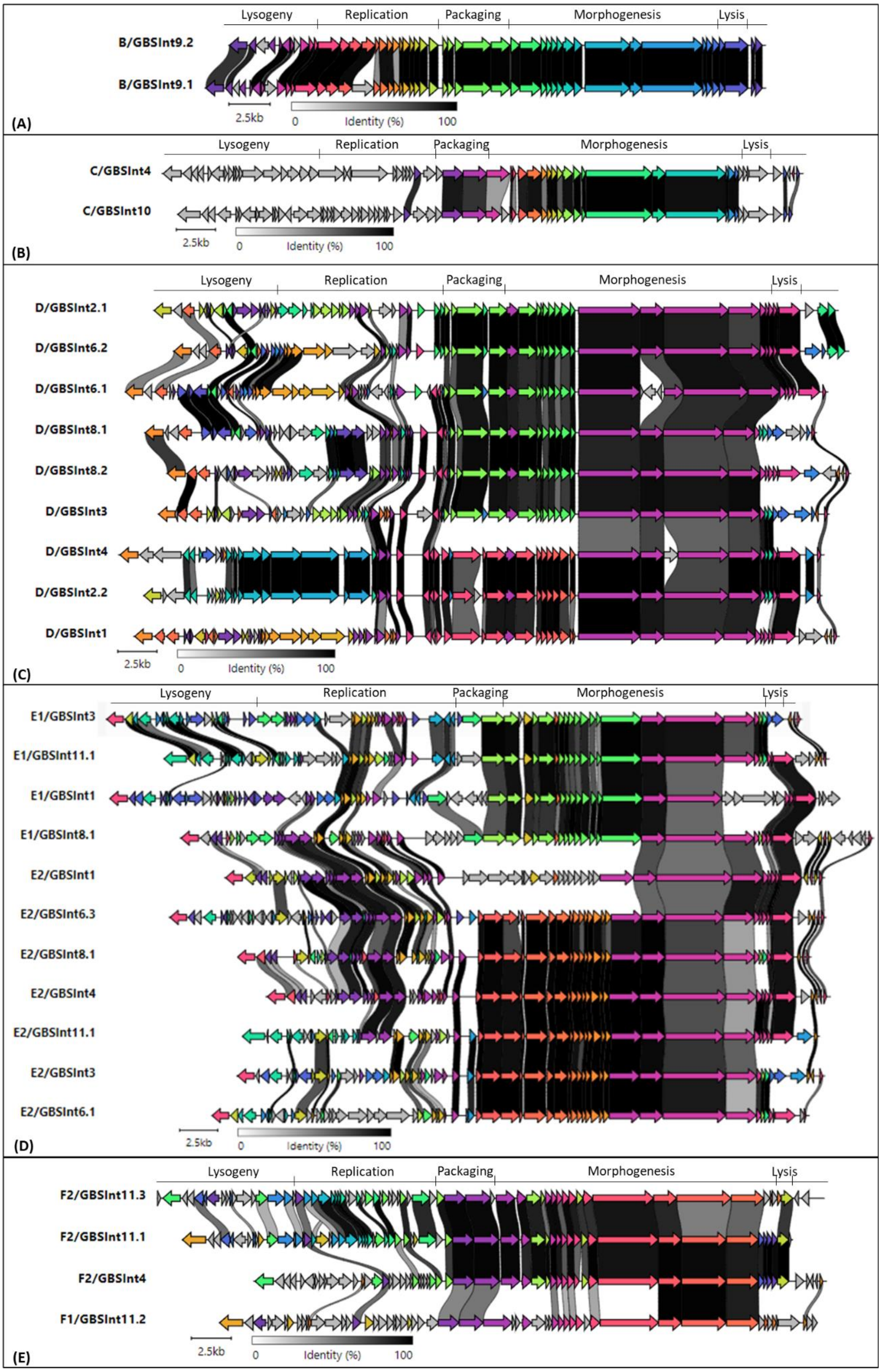


**Fig S4. Comparative analysis of phages belonging to the same prophage group but with different integrase types.** (A) Prophage group B. (B) Prophage group C. (C) Prophage group D. (D) Prophage group E. (E) Prophage group F. Genes were automatically coloured by Clinker according to their homology. Genes with more than 40% of identity are linked with gray-black strokes, as shown in the scale.

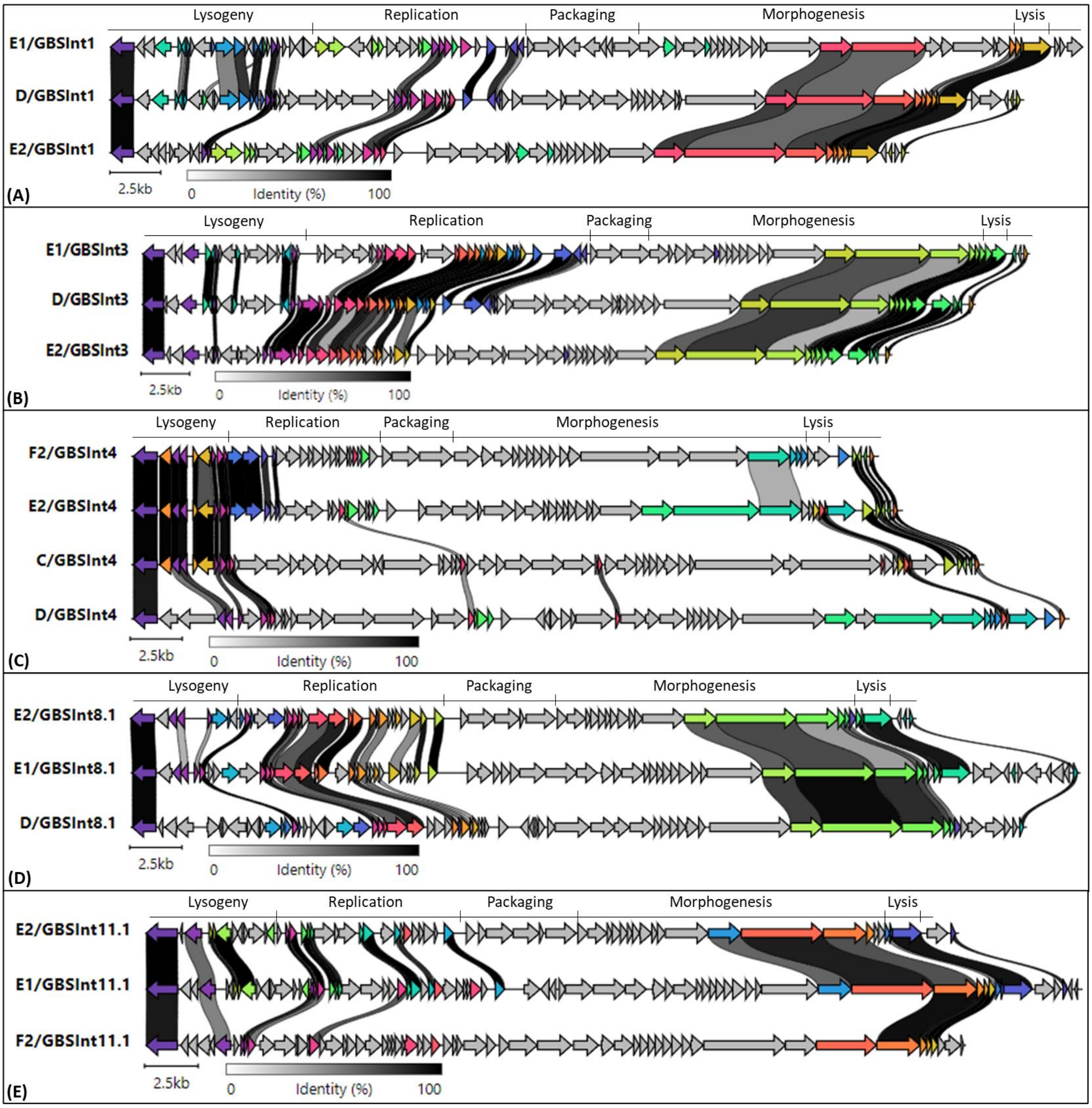


**Fig S5. Comparative analysis of phages with the same integrase type but belonging to different prophage groups.** (A) Integrase type GBS*int*1. (B) Integrase type GBS*int*3. (C) Integrase type GBS*int*4. (D) Integrase type GBS*int*8. (E) Integrase type GBS*int*11.1. Genes were automatically coloured by Clinker according to their homology. Genes with more than 40% of identity are linked with gray-black strokes, as shown in the scale.

# Supplementary Data

**Data S1.** Prophage-group database for the screening and classification of prophages by prophage group.

**GroupA-*hha*I**
ATGACTCTAACCTTTCTTGATTTCTTTGCAGGAGTGGGTGGCTTTCGTCGTGGGTT
GGAATTGGCAGGTATGACCTGTCTTGGGTATTGTGAGAAGGATAAGTTTGCACGG
AAATCCTATGAAGCAATGTACGATACAGAAGGAGAATGGTTTCATGACGACATC
ACAAGCATTGACCCCACACGACTTCCAAAAGCAGATTTATGGACTGCGGGAAGC
CCTTGTCAAAATGTGTCTATCGCAGGAAAGCGAGCCGGCCTATACGGTGAGCGA
AGTGGACTCTTTTTTACATTTGTTGACCTCATCCAAAGCCAAAAGGAAGAAGATA
AACCCGAATGGGTTCTCCTTGAAAATGTTAAGGGACTTCTATCAAGTGGCGGGG
GACGAGATTATCTCGACTATCTCTCTATCTTGGATGAAGCAGGGTACGACCTTGA
ATGGCAAGTGTTCAACTCAAAAGACTACGGAGTTCCCCAAAACCGAGAACGTAT
CTACACTCTCGGACATCTTAGAAGTCGAGGTCGACGACAAGTACTACCTCTCAGC
GGAGAAGGCGGTAGCTATCTTAAGCAACTTGTAGGTGGCATGCAAAGCTATCGA
GTCTACGACCCAAGTGGCATTGCCACAACCCTTGTTGGTGAGGGGGGAGGACTA
GGAGCTAAGACAGGTCTTTATCTGATTGACCAGTCTTTGACCGAGCCAAAACTAA
CTGAAGAAGCACGGTGTATAACCGCTCGTTATACGGCAGGAGCTACAAAAAGAA
CAGCAATGAACTCTGGTGTTCTTGAGGTTCAGCCCATTCTGACCCCAGATAGAAT
CACTAAACGCCAAAATGGTAGACGGCTAAAGGAACAGGATGAGCCTATGTTTAC
TCTGACTTCTCAAGACCGTCACGGTGTCCTTGAAGGCATCAAGGTCAGAAATGGG
ACAAAGCAGGACTACCAAGTCGCAGAGGTTGGCGACTCTGTTGACCTTTCCTATC
CAGGCTCACAGACGAGACGAGCCAGAGTAGGGAAGGGCATCGCACACAACCTCT
CTTGTGGTGGTCAGATGGGTGCCGTGGTTTGGAAGGGTCGAGTGGTGAAAATCA
GACGTTTGACCCCACGAGAGTGTTTCAGACTCCAAGGGTTTTCAGATGACTTGTT
TGACAAGGCTCAGGCTGTTAACTCTGATGCCCAGCTCTACAAACAGGCTGGCAAT
GGCGTGACAGTAACCGTGGTTTATGCCATTGGGAAAGCTATTTTAGAAGCCAATA
AATCTGCAAATAATAAGCAGAAATGA

**GroupA-*clp*P**
ATGCGTAAATTTTGGAATTTTACTGACGAAGGAGAAGTCCGCACCCTTCGGATTG
AGGGACAGATTGCGGACGAGACTTGGTTTGGGGATGAAGTCACCCCGCAGCTCT
TTAAGAATGATTTGTTAGCAGGAACAGGCGACATCACCCTCTGGATAAACAGTC
CAGGGGGTGATGTATTTGCGGCCGCTCAAATCTATAACATGCTTATGGATTATCA
GGGCGATGTCCATGTCATCATTGATGGTCTAGCCGCCAGTGCTGCCAGTGTCATC
GCCATGGCAGGGACAACAGTTTCCATGAGTCCAGTTGCCATGATGATGATTCATA
ACCCTTGGACGTTTGCGCAAGGTGAAGCGAAAGATATGGCCAAGGTCATTGAGA
TGCTTGGCGAAATTAAGGAGTCCATTATCAATGCCTATGAGCTTAGAACTGGACT
TTCCAGAACCAAGATTTCTCATCTTATGGATTCGGAATCTTGGTTCAATGCCAAG
AAAGCTGTGGAGCTTGGTTTTGCGGATAAAGTGCTCTTTGAGAAAGAGGAGACA
CCTGAGCAGGATCATCAAAATAGCTATACCTTTAGTAGAGTGACTGCTGCTCATG
ATTTGGTGGTGAAACTGCAAGCGAGCCTTCAACCGCCTAAACCACAGAAAACAA
TCCCTTTCAATCAGTTGGAAAAACGATTGAACCTATTGAAATAA

**GroupA-holin**
ATGAAAGAATTACTGGCAACAAATAAGGTTCTCTTTTCAGCAATCGGAGGACTTA
TCGGTTCGATTTTTGGAGAAGTGGATGGGGTCTTATATGCCCTTTTTATTTTTCTC
ATCATTGATTATGTGACTGGAGTCTTTGCGGCAATCGTCGAGAAGAATTTATCAA
GCGGTCTTGGTTTCAAAGGCATCTTTAAAAAGATTGCCATTCTCTTTTTGGTATCC
GTGGGACACCTCATTGATACTGAAATCATTAAGCAGGGTGGAGCGATTCGCACC
ATGGTTATTTTCTTTTACTTGAGCAATGAAGGTTTGAGTATTTTGGAAAATGCGGT

GCGGATTGGCTTGCCTATCCCTGAGAAACTACGAGCCATCTTAAAACAATTCAAC
GAAAAAGAAGGAGACTGA

**GroupA-lysin**
ATGACTTTTTTATCGAAGATTAAAGACGGTTGTTTAGCGTCTTGGGAGCACGGTA
TTCTGCCTTCCGTGTCAGCAGCACAAGCTATCTTAGAGAGTGGTTGGGGAGAGTC
TTTGTTAGCACAATACCCTATTCATAATCTCTATGGAATCAAAGCTAGTTCTGATT
GGAAGGGAAAACGTGTTGACCTTCCAACTCAAGAATTCATTGATGGGAAATTCG
TCACGGTTGCCGCAACGTTTAGAAAGTATGACTCATGGGAGGAATCCATTAAAG
ACCATGCCTTATTTGTTTCTGAAAACGATTGGCGACGATTACACTATCAGAACGT
GCTTGCCGAAGAAGATTATAAAAAGGCCTGTCTCGCTTTACAGGTGGCAGGTTAT
GCGACAGACCCTCAATATGGGTCAAAATTGATTACACTTATTGAAACGCACTAG

**GroupB-minor_tail_protein**
ATGACTGAAACGTTTGAAGGCTTATACGTCAAATTTGGTGCTAATACTGTTGAAT
TTGATAGGTCTGTAAAAGGTATCAACACTGCCTTATCTAGTTTGAAAAAAGACTT
CAATAACATCAACAGACAATTGAAGATGGATCCAGACAATGTTGACTTGTTGAA
TCGTAAGTTGGTTAACTTGCAAGAACAGGCTCGTGTTGGTGCTATGAAAATTGCT
GAACTCAAAAAGCAACAGAAGGCACTGGGAGAATCTGAAGTTGGGTCAGCACA
GTGGAATAAGCTTCAACTTGAAATTGCTAAGGTTGAATCACAGATGAAGATTGTT
GATAAGGCAATGGAGTCAACAAAGAAACACATTGAAGATGTAGGAGACCCAAA
GTCTATTCTGAATCTTAACAAAGAACTTGATAATGTTGCTAAAGAGCTTGATATT
GTCAATCAAAAGCTTGAGCTAGACACTGACAATGTCGAACTAGCAGAGCAAAAA
ATGAAACTACTTGGTAAACAGTCGGAATTGGTTGGGGATAAAGTCCAAGAATTA
AAGAAAAAACAAGCTGCCCTTGGCGATGAGAAAATAGGTACAGAAGAATGGCG
TCAACTTCAAAATGAAATCGGTCAAGCTGAAGTTGAAGTTCTAAAGATTGACCGT
GCAATGGACATTCTTGGTGAGTCAAGCCGTTCTGCAACTGGAGACATCAAAGAG
GCAACCAGCTATTTAAGAGCTGATGTCATGATGGATGTTGCAGATAAGGCTGGTC
AGATTGGCCAGAAAATGGTTGGCGCTGGGAAAATGACAGTAGATGCTTGGTCTG
AGATAGATGAGGCTCTGGACACCGTCACAACCAAAACTGGTCTGACTGGTGATG
CCTTAGCAGAGCTTCAGGAAATTGCTAAAGACATTGCTACTGGTATGCCTACCAG
CTTTCAGAATGCTGGTGATGCCGTTGGGGAATTGAATACTCAGTTCGGTTTGACT
GGGGAAAAGCTGAAATCAGCATCTGAATTACTTATCAAGTATGCTGAGATTAAC
GAAACAGACATTTCAAGCTCTGCCATTTCTGCAAAACAAGCTATTGAAGCTTACG
GTTTGACAGCTGAAGACTTGGGAATGGTCTTAGACAATGTGACCAAAGCCGCTC
AAGATACAGGACAGTCAGTTGACACGATTGTTCAAAAAGCCATTGACGGTGCTC
CTCAGATTAAAGGTTTGGGACTTTCTTTTGAAGAAGGTGCTGCACTGATCGGTAA
GTTTGAGAAAAGCGGTGTGGATTCATCTGCTGCTCTATCCTCTCTATCGAAAGCT
GCTGTCATCTATGCTAAAGACGGTAAGACTCTGACAGATGGATTGAATGAGACT
GTTAGTGCTATTCAAAATTCTACTAGTGAGACAGAGGCTTTAAGTATTGCCTCAG
AAATCTTTGGTAGTAAGGCTGCTCCTAGAATGGTCGATGCTATTCAGCGTGGTGC
TTTTAGCTTCGATGACTTAGCTGAAGCAGCTAAAAGTTCCTCTGGTACTGTCTCC
ACCACATTTGATGAGACGCTTGACCCAATAGATAAGTTGACTCAGTATTCTAACC
AAGCAAAAGAGGGAATGGCAGAACTTGGCGGTAAATTGCTTGAGACTGTCATCC
CAGCTTTAGAACCTTTGATGGGTATGCTTGAATCTTCTGTCAATTGGTTTACTAGC
CTAAACGAAACTGATCAACAGACTATCGTGATTCTTGGCCTAGTTACAACTGCTG
TGATGATGTTGCTTGGTGCAATTGCACCGCTGGTCATCGCCATAGGGGCAATAGG
TGCGCCTGTCGGAATTGTAGTGGCGGCAATAGTAGGGGCTATTGCCGTCATAACA
CTTATCATCCAAGCAATCATGAACTGGGGAGCCATAACTGAATGGCTTCAGTCAA
CGTGGGATTCTTGTGCTGCCTGGCTTTCTGAATTGTGGACTAACATAGTCACGAC
TGCCACCACAGCGTGGTCAAATTTCACTGCCTGGCTTTCTGGCCTTTGGTCTTCAG
TAGTCTCAACTGGACAGTCTTTGTGGTCTAGCTTTACTAGTTCCTTGTCCAATATT
TTCTCAAGTTTGATTACAGGTGCTCAGTCTCTGTGGTCAAGTTTCACTTCCACTCT
TTCCAATTTGTGGTCTGGACTGGTCTCAACTGGGTCAAATTTGTTTAATAATTTGA

GTAGCACGATTTCAGGAATTTTTAATGGGATACTTTCAACAGCAAGCAATATTTG
GAATTCCATAAAATCCACTATTTCCAATGCAATAGATGGGGCGAAAAATGCAGT
GTCCAACGGGGTCAATGCCATCAAGAATCTGTTTAACTTCCAGATTAAATGGCCT
CATATTCCACTACCTCACTTCCGTGTGAGTGGTTCTGCTAACCCTCTGGATTGGCT
AAAAGGTGGCTTACCAAGTATCGGCATTGACTGGTATGCCAAGGGCGGTATCAT
GACCAAACCAACCCTATTTGGCATGAATGGAAACCGTGCAATGGTTGGCGGTGA
GGCTGGCGCTGAAGCCATCTTGCCATTGAATAAGTCAACCCTGGGGGCAATTGGT
CAAAGTATTGCTAACACGATGAATACATCGAACAATATTAACGTTAACTTCTCTG
GCGTCACTATCAGGGAAGAAGCTGACCTTAACAGACTAGCCAACGTGGTTGGAA
ATCGTATTGCTGAAGAATTGCAACGTAAAACTAATTTGAGAGGAGGAATGGCAT
GA

**GroupB-hypothetical_protein**
ATGAAATACACTTATTTAGCATTGTTTGAAGTGGATAAAGAAAACGGTGGCTATA
ACATTTCTTTCCCTGATTTTCCTGGAGCATTTAGTGAAGCTGATAGCTTGAACGAG
GCCATTTTTAACGCTCGTGAAGTCCTTGAAATCTATACAATTATGTTTGAAGACG
AGGGCAAAGAGTTTCCTAAAGCATCATCATTCAAGGCTCTTGCAAGCAATTTAGC
AAGTGACGAAGATGTGATTCAGGCTATTTCAGTTGATACTGAGCTTGTCCGTGAG
CGTGAACGCTCTAAGATTGTCAACAAGACTGTCACACTGCCAAGCTGGCTTGTGG
AAGTTGGAAAAGAAAACAAGGTCAACTTTAGCCAGCTGTTGCAAAAAGCAATCC
GTGAGGAATTGCAAGTATAA

**GroupC-repA**
ATGGCAGAGAATGATTTTAATTTGCTACCGTTGCTGGATTATATCAATCCTGCCA
CGGTAGACTATCAAACATGGGTTCAAGTAGGAATGGCCCTAAAGCATGAAGGTT
ATACAGCAATGGATTGGGATGTTTGGTCACAATCTGATAGTAGATATAAAAAAG
GTGAGTGTTTTGCAAAATGGGATAGTTTCCAAGGCAATGGCTTTGGGACTATCAC
AGGGGCAACAATCACACAGCTAGCTAAAGATAATGGATGGACATCATCAGAGTA
TCGTAACAGCGATGATGCTCATGAACTCAGCTGGGACGATACAATCGATCGTGA
TTATAAGATTGTCGATAAAAATTGGATTGAATCGAAAGAGATTCAAGAGCCAAG
AAACTGGAATCCAGTTCAAGATTTAATTACTTACATAGATACCTTATTTGAATCA
ACTGACAAAGTTGGTTATGTAACAGAGACCTATCCAATTACCCTTGATACGGGAG
AGATTGTTTATAAACCAACAAAAGGAGCGTATGACAGGACTGCTGGTCAACTGA
TTGAATCACTCCAAAAAAATCCTACTGACTTAGGAGCAGTATTTGGAGACTTCAA
AGAAGAGGCTGGTGCATGGATTCGTTTTAATCCACTAGATGGAAATGGTGTCAA
GAATGACAATGTAACAGACTTTAGATATGCCTTGGTTGAATCCGACAGCATGGA
ACTTGGTAAACAGTATGCTTTGTTTAAAGAACTAGAATTGCCAATAGCAACCTTA
GTCCATAGCGGTAAAAAATCATTACACGCTATTGTCAAAGTAGATGCTCGTGATT
ATCAGGAGTACCGCAAACGGGTTGATTATATCTATCAAATTTGTAAAAAGAATG
GACTTGATATTGACACACAGAACCGCAACCCTAGTCGATTATCACGCATGCCTGG
TGTGACTCGGAATGGGCACAAGCAATTCTTGATTGATACTAATGTGGGTAAAACC
AACTACGAAGAATGGTATCAATGGATTGAAGATTTAAATGACGATTTGCCTGACC
CAGAGACGCTAGCTGACGAATGGGATAATATGCCAGAATTGGCACCGGAACTCA
TCAAAGGAGTTTTGCGTCAAGGCCACAAGATGTTGATTGCTGGACCATCAAAAG
CTGGTAAGTCGTTTGCATTAATTGAGCTATCCATCGCGCTGGCTGAAGGTAAAGA
ATGGTTAGGCTGGCAGTGTGAACAAGGTAAGGTCTTGTATGTCAATCTGGAACTG
GATAGACCGTCAGCTTTGCATCGCTTCCGTGATGTATACGAAGCTATGAGCTTGC
CACCAGCAAACATCAAGAATATTGATATTTGGAACCTACGTGGAAAGACCGTTC
CCATGGACAAGTTAGCGCCTAAGCTTATCCGTCGTAGCTTAAAGAAAAATTACCA
AGCGGTCATCATTGACCCTATCTATAAGGTGCTGACTGGTGATGAAAATAGTGCG
GACCAAATGGCTCACTTTACCAATCAGTTTGACAAAGTAGCTACTGAGTTAGGTT
GTAGTGTGATTTACTGCCACCATCACTCAAAGGGGAGCCAAGGCGGTAAAAAAT
CTATGGACCGTGCAAGTGGTTCAGGAGTGTTTGCCCGTGACCCTGATGCACTGAT
TGACCTAGTAGAGCTAGAATTGACTGAGGAACTCATCAAGTCACGCTCAGAAAA

AGCAGCCGCTAAGATTTACCAACAAGCCTTGCAAGAAAAAGCGCTAGCCTACTA
TCAACAGGAAGTAACGCTAGATGATTTGGAAAGTCGTTATCAGATGCAGCAACA
TTTTGACAAGGCTATCAAGGACATCATGATTAAACAGCCCTATCTGGAAGCGGTC
AAGAAAGCCCAGTATGAGGTGGAGATTTCCACTGCCTGGCGAGTTGAAGGGACT
TTACGCGAGTTTGCTAAATTCCAACCAGTTAACATGTGGTTTAGCTATCCAGTGC
ATGATGTAGACACAACGGGTGTCTTGGCTGATATATCACTAGAAGATAATGTGCC
GACTTGGAAGAAGAATTTTGAGAAGAAGAGTCCAAGAGAGTCCAGGGAAAAGA
AATCTCAAAAAGTAGAGACTGCAATTAATTCATTGAATGATGGAATAGAGCCAG
TCACAATCGATAACTTAATCGAATATTTTTCTACTGAAGATAAGCCAGTTTCTGA
AAAAACTATTCGTAGATGGATAAAAGAAAACGGTAAATTTGAAGTTAAAAATAA
ACAAATTTTACCAATAGAAGAACCTAAAACCAATAAAAGTTTGTGA

**GroupC-terminase_large_subunit**
ATGACGACTAAGACATGCCCTAAGATAAATATTGTTATCAATCACCCAAGCCGAGTTTTTAATCGTCA
CATCTATGACAATCTATATAACTACGATAACTTTACCGAAGTACACTACGGCGGTGCGTCAAGTGGT
AAGTCTCATGGTGTTTTTCAAAAGATAATCTTAAAAGCACTTAATCCTAAATTTAAACATCCTAGAAA
GATATTAGTCCTTAGAAAAGTTGGTGCAACAGTGAGAGATTCTGTCTTCGCTGATATCATGTCTAACC
TGTCGTATTTTGGCATATTATACAAATGTAAGGTAAATATGTCAGCGTTTAGAATAACGCTTCCTAAC
GGCTCAGAATTCATATTTAAAGGTATGGATAACCCAGAAAAGATTAAATCAATTAAGGGTATATCCG
ATGTTGTCATGGAAGAAGCTAGTGAGTTTACTCTTGACGATTACACACAGCTTACCTTACGTCTTAGG
GACAAGAAACATTTAGAGAAACAAATCTATCTTATGTTTAACCCTGTGAGTAAAGCAAATTGGGTTT
ATAATGCTTTCTTTGTAAAGTCTCCAAAGAACACAGTCGTCTATCAAACGACTTACAAAGATAATAGA
TTTCTTGATGAAGTTACTAGAGAAAATATCGAGGAGCTAGCCAATAGGAATGAAGCCTATTATAAGA
TCTATGCGCTTGGGCAGTTTGCTACACTTGATAAACTAATTTTTCCCAAATATGACAAGCAAATATTA
AACAAAGACAAGTTATCACACTTGCCTTCTTTTTTTGGTTTGGACTATGGGTTTATCAATGACCCTTCG
GCATTTTTGCATGTTAAAATCGATGACACAAACAAGAAGTTATACATCTTAGAGGAATATGTCAGAA
AAAATTTGACAAATGACAAAATAGCAAATGCTATAAAGGACCTTGGCTATGCCAAAGAAGAAATCA
GAGGAGATTCGGCTGAAAAGAAATCTAACCAAGAGCTGAGGAATTTAGGTATTCCTAGAATGATTG
ATGTTGCCAAAGGGCCTGGAACCGTTATGCAAGGAATTCAGTACCTGCTTCAGTATGATTGGATTGT
TGATGAAAGGTGTGTCAAGACTATTGAAGAACTAGAAAATTACACTTGGAAGAAAGACAAGAAGAC
TAATGAGTATATCAATAAACCAGTTGACAGTTACAACCACTGCATTGACGCCATAAGATATGCCGTA
CAAGACAGAATATACCAGTCGGCAGATAGAAGTAAGCGCATGAAGAACGCGAAGTACTATTTTTAG

**GroupD-terminase_small_subunit**
ATGGGGAGAAAATTAAAAGTAGTTGAAACGACAAAAAAACACCTTACTAAAGA
AGAGAAAAAAGTTCGAGAAACCGCTCAAGAAAAGGCTTCTGACGGTCTTGCGAA
ATTGCAAGTAACGCCACCGCGGCACTTAAACGAAGTGGCTAGAGCTGAGTATAG
AAGAATTATAAATGACCTGCAGACCCTACCCACAAGAAACTTAGATAGAGGGCT
ATTAGAGTTATATTGCACATGGTATGCTATCTACAAAGAAACAAGTAGAAAATTA
GATGAAGTTGGCTATTTTGCGAATGATCCAGACAAAGGTTTAATTCCGAGTCCGC
TTATTTTAACACTGGAAAAGGCTACAACTAATATCAGAAGTAGTGCTAGCCAATT
AGGTTTAACAGTTGACAGTCGTATGAAGATGTTTATTCCTAAGGAAGAAGAGAA
ACAAAAAAGTATTTTTGATAAATTTGGAGGGTAA

**GroupD-terminase_large_subunit**
ATGACAATAGAATACGATTATTCAGCAATTAGCGACATTTATAAAGATGACACTT
TTTATTATGCAAAAAAGATTGTTGATGAAGAGATTAAAGCAAGCAAGAAAGTGT
TTAAGGCTTGTTTAAGACATTTGAATGACCTCAAACGTATAGAAGATGAAGATTT
TAAATTCATCTACTTGCCACAAAAAGCATCTGATCCAATTAATTTCATTGAGATTT
TACCAGACGTCAAAACAGGAAAACCTTATCCATTAGCAATGTTCCAAAAATTTAT
TATTGGGAATTTATATGGATGGCGGAAGAAAACAGACCATTCCTTAAGACGTTTT
AGAAAAGCTATGATTTCTGTTGCTCGTAAAAATGGTAAAACCATTCTTATAGCTG
GTATATTGCTTTATGAATTTTTGTTTGGTCACAATCCGTCTATGAGTAGGCAATTG

TTTTGTACAGCGAACGACCGCACGCAAGCTAAAATAGCTTGGGATATGGCAAAA
AAGCAGTTAGCTTCCTTAAGAGCAAAGGATGCCGATGTCAGAAAAGCTACAAAG
ATTGTCCGTGATGAACTAAAAAACTTACATGATGAATCTTATATAAGGGCGCTTA
GTCGTGATACTGGCGCAGTTGATGGATTTGAACCGTACGTTGGAGTGTTGGATGA
GTTCGCAGCGTCAAAGACAAACGAAATGTTAGAACTATTGGAATCTGGTCAAGG
ACAGTTGGATAATCCGTTTATCTTAATCATTTCAACGGCTGGTATGGATTTGAAT
GTTCCGATGCACACAATTGAGTATCCATACATTACTAAAATACTAGACGGAGAA
ATCACAGACGAGGGCTATTTTGGCTATGTCGCAGAGCAAGACAACGAGGAGGAA
ATTAAAGATGAAACGAATTGGATAAAATCTAATCCAATTCTCGAAGTCGATACTC
TACATGATAAGTTGATGGACTATCTAAGAACTCGTCGTAAGGTATCTCTAGAGAC
TGGAGAAGTCAACAAAGTGTTGATCAAAAACTTCAATATGTGGCGTCAATCCAG
CGAAGAATCATATATAGATAAACAGTCGTGGGAGCTTGCTAAGATTGATAAGCC
AGACACATACAAGCGTAGGGTTTGGCTAGGTGTTGACGTTGGGCGTGTAAGTGA
CTTGTTTGCCATTAGTCCTGTTGTTATGATGGATGATTATTGGTATGTTGATAGTT
TTTCATTTGTAGCTACAAAGTATGGCTTAACTGCCAAAGAAAAGCGAGATGGTGT
ATCTTATAGCAATCTAGAACGTCAAGGATATTGCGAAATAACAACCCTTGAGAG
CGGGGTTATAGATGATGAACGGGTTTTGGAAAAAAATAGAGGAGTTAATCTATAT
AAACGAATGGGAAGTACATGGGATTTGCTTTGACCCATACCAATTCGGAACACT
ACTTACAATGATTGAAAAAAGACATCCGGAATGGCCTCTAATAGAAGTTTCGCA
AACGACAATGGTGTTAAACATGCCGACAAAACAATTTCGTGACGACCTTAAAAA
AGGCAAAATAAAGCATTCTGGCAATCCACTATTGACCATGGCTGCTAACAACGC
TTATATTAAAACCGATAACAATGGCATGAGGATTGACAAGAACAAGAATAGCAA
CAAGATTGACCCGCTAGACGCAGTTCTTGACGGTTATGCTGTATGTTACCTAGAA
CCATTTGACGGTTCTGGCTACTGGACAAGCGAGAAAATTTTAGGAGGAGAGACG
CTATTTTGA

**GroupD-phage_major_tail_protein**
ATGCAAGCAGTAGGATTTAAACGAATGACTATCCAGTTATTATCTGAAAAAAAA
GACAAAATTGTTATCGAAGGTGCATCAGGAAAAGGTGCTACAAAAACAGCTAAG
ATTAGTGGATTATCAGCAGCTCCTGTCAAAACTTACGGTTCTGATATCGCTTATTA
CACTTCTCGTCGAGGTGTTGGCGATGTAAAAATGGAGATGGAAGCAATTGATATC
CCATTTGACTCGCTTAAAAAAGTATTGGGATATAAAACAGGAACCACCTCAACA
GGTGTTTCTTTTATTGGAGAAGATACAGAAGCTCCAGAAGTATCAGTCTTACTTG
AAGCACCTGGAACTGAAGGAAGCGTTTATCTAGGCTTCTTCAAAGGAACTTTCTC
GATGGAAGATTTCGAATTAAAAACACAGGAAGAAAAACAGGATGGTTTAGACTC
TCAAAAATTAGTATTCACAGCACAACCAGGAGATACTGGAGAAGCGAAAGGTCA
ATATGTCGGCTGGGCAATAGATAAAGAAGCAGAAGCTAAGGGCGAAAATGCAA
AAGCGTTGGTTAAACTTTTGAATCAAGACGCACCAGGAGTGTAA

**GroupE1-tail_protein_2**
ATGGTCACAAGTTCACCGATTTATGGGAAAGATAATATTTTGATGTTCCGTGTGC
TTGGAGATAAGGCAGCTGCGGCTAAATTGTCTTTTCAGACAGAACATAAATGGA
AATATAGTAGAAAAACAGATGCTAAAATCACAAAAGATGGTGCTATCAATTCTG
ACAAAGGGCTTGAGGTAACTTTGGAAATCAAAGGCGTGGCAAGTCGTGATGAAT
TGAATACGACACTAAAGAATGCTGTTTTAGAAGGTAAACAAATTGAAGTTTGGG
ATATTGACTTGAATAGCAATAACGATTCAGACGGGAAATATGATGCAGAATATG
CTATTGGGCGATTGGGATCTTGGGAAGTCCCTTCCAATGTTGAGGAATTTTCTGA
AATTTCCACAGAAATGGCTATCGATGGCAAACCAGTTAAAGGTAAAGCCACTCT
TACAAAAGAACAGATTAAAGCTATTCAATATGTATTTAAAGATGTAACAAAAAT
TGATGAATCTTCCGTTTCTAGTCCGGGTTCTGTTCATAGCAGATAA

**GroupE1-capsid_protein_E**
ATGGCATTAATTTATGACGTTGTAACATCTGCTAACATCAAAGGATTTTATGATA
AACAACAAGCAAATGTTGACTTGACTTTGGGAGAAAAAGCTTTTCCATCTAAAC
AACAACTTGGTCTTAAGTTATCATTTATCAAAGGAGCAGCTGGTAAACCAGTTAG
TATCAAAGCGGCAGCGTTTGACACTAAAGTTCCACTTCGTGACCGCATTGCTGTA
GTCTTATTAGACGAAGAAATGCCTTACTTTAAAGAAGGTATGCTTGTAAAAGAGG
CTGACCGTCAACAACTTAACGTTTTAGCGCAAACTAAAAATCAAGAACTTATTGA
CACAGTGTTATCAACAATCTTTAACGATGAAACTACTCTAATCGCTGGTGCTAAA
GCACGTCTTGAAGCTATGCGTATGGAAGTGTTGTCAAGTGGTAAAATCCACATCA
ATTCAAATGGTGTTATGAAAGATATTGATTATGGATTAACTGGAACTCAAACGAC
TAAGAGTGAACAAAAATGGTCAGAAAAAGACACCGCTAACCCTCTTGCTGATAT
CGAGAAAGCTATTGAAACAGTAACAGAGCGAGGTCACGTTCCTGAAGCCATCGT
CTTAAACTCAAAAACTTTTGGCTATATCAAAAACGCAAAAGCAACCGTAAAAGC
AATTAAACCACTTGCACCGGAAGGCTCAATTGTTACTAAAGCAGAATTAAAATCT
TATCTTTCTGAAGAATTGGGATTAAATATCTTACTTAAAGATGGTGTGTTTGTTAA
TGACGCAGGTGAAAGCAAGAAGTATTTCCCTGATGGCGTAGTTACACTTGTACCT
AATGGAAATCTTGGCTATACAGTATTCGGGACAACTCCAGAGCAGTCTGACCTTA
TGGGTGGCCAAGCAACTGATGCACAGGTATCTCTTGTAGAGACAGGTATTGCTGT
TACAACTACTAAGACTACTGATCCTGTTAACGTACAGACTAAGGTTTCTATGATT
GCTCTACCATCATTCGAGCGCTTAGATGAAGTACAGATTGTAACAAGTTCGGAAG
TATCATTATAA

**GroupE2-phage_major_tail_protein**
ATGGTAGCAAATTCATCAAACGTTACTACGGCTAAACCTAAAATTGGTGGTGCTA
TTTATACTGCGCCGCTAGGAACAGAATTACCCAAAGACACAGCATCAGAGTTAA
ATGAAGCCTTTAAGTCATTAGGGTACATTTCCGAAGACGGCTTATCAAACGAAG
ATAAACGAGAATCAGAAGAGATCCAAGCGTGGGGTGGCGATGTTGTAGAATCTG
CACAAAAAAGTAAAGCAGATAAATTTACATATACATTGATTGAAGCATTGAATA
TTGAAGTACTCAAAGAAATCTATGGCAAAGATAATGTAACTGGAGACCTTAAAG
CCGGGATTACTGTTAAATCAAATTCAAAACCACTAGAGGAACATTGTTTGGTTAT
CGAGATGATTTTGAAAAACAATACAGTTAAACGTATTGTAATACCAAAAGGGAA
AGTATCCGAAGTTGGTGAAATTAAGTATGTCGATAACGAAGCGGCTGGTTATGA
GACAACGTTACAAGCGTTTCCTGATGCAGAAGGAAACACTCACTATGAATATAT
AAAAGGAGCTGGTTAA

**GroupE2-phage_protein**
ATGAGATTTGTTAATTTTGACTTAGTAACCCCACAAAAAACGGGAGAAAAAGAC
AGACTCGGCAACGACATCACGAAAGATGTTGTCAAAAGAGTTGCTAAAGGTCGT
TTTACTGAATGGTCGGCTGATGACGTGTCCTTATACGGTCGAGATTTAACGTCTA
GCGCACGCAAATTGCTGACTAATCAAGTTAGCAAGGCGGAAGCCAAACAAGCGT
CACACGTTGTAATAGACGGCTCGAAATACAAAGTAGAATCCGTTAAAGACCTTG
GTAGATGGAGACTACTCGTCATTAAAGGGTATCGCTTATGA

**GroupF1-structural_protein_GP20**
ATGAGCCTTAAACGTGAGATGTTGGTTGCCGCAGGTATCACAGATAACAGTGTG
CTGGATAATATCATGCAAGCGTACGGTGCAGGTATTGAAAACGCAAAAGCACAG
GCTAAGTCAGAGTTACAGGCAGAAAACGACACCTTGAAACAACAGCTTGGGCAA
CAAAACCAAGCTATCAAGGATTTACAGGAAAAAGAGGGAGCAAGCGAGGAAAG
CAAGCAACAACTGGCAGACCTACAAGCCCAATTTGACCAGTACAAGACTGATAG
TGAGGCACAGCTTGCTCAGGTTACTAAAACTAACGCTGTAGCCCTTGCCTTGAAA
GATGTGGGAGCTTACAACTCTGAGGACTTGATGAAGTTTATTGACCTAGACAAGA
TTGAACTAGGCGAAGACGGAAAACCTCTCTTAGAGGACACAATCAACAGCCTCA
AGGAAACTAGCCCTTACTTATTCCAAGGCGAAGATAAGCAGCCTAACCCTAACA

TCTCTGTACCAGGTAACCCAGCGGCTGACAATGGGGACAACTTGAGTGCAGAGG
ACAAAGCCCTTTTTGCCGGCTTTGACAGCGTATAA

**GroupF1-terminase_large_subunit**
ATGGCGATACTGAACCTAGCAAAGCTGATTAACCCAGTATTTGATGAAGTGCTCT
ATACGCTCAAGAGTCATATAGTGCTAAAGGGTGGCCGTGCCTCTACTAAGTCCTC
TGTGGTGTCTATTGACCTAGTAAACGACTTTATCAGTGACCCTCTGGGTAATGTG
GTAGTCCTACGGAAAGTGGGGAAGTATCTTAGAATGTCTGTCTACGAGCAGATA
AGATGGGCGATTTATGAGATGGGGCTAGCCAATCAGTTCAAGTTTGGTAAGTCAC
CGCTACAAATCACTCACAAAAAGACAGGAACAGCTTTCTACTTCTACGGCGTAG
ATGACCCAATGAAACTCAAATCACAGAAGATAGCCAAAGGGTATGTCATGTCTG
TATGGTTTGAGGAACTTGCAGAGTTTGCAGGTCGTGAGGACATTGATATAGTTGA
GGATACCTTTATCCGTCAAGAGTTGCCAAATGGCAAACAGGTCAAGGTTTATTTT
ACATACAACCCGCCTAGAAACCCCTATGACTGGATAAATGGCTGGGTGGCTGAG
AAAGCTAGTGACCCAACGTATTTAATACATCACAGCACCTACCTTGATGATAAGT
TAGGCTTTTTGTCTAGGCAGATGAAAGAGAAGATAGAGCGGTACAAGGAGACTG
ACCCTGACTACTATCGCTGGATGTACCTAGGAGAAGTTATAGGTCTTGGTAATCA
TGTTTATAACATGAACTATTTAAACCACTTGAGAGCCTCCCTAATGATGACAAG
GTGATAGGTATATCATTTGCTTTAGATACTGGACACCAGCAATCGGCTACAGCCT
GTGGAGCTTATGGATTGACTGCCAAGGGTAATGTTATCTTGCTTGATACTTTCTAC
TATTCACCAGCTGGCAAGACGATTAAAAAGGCACCCAGTGAGCTCTCAGTAATG
ATACATGACTTTATTGATAAGGTCATGAAGACTTACAGAGTGCCAAAGCTCAAG
ATGACTATTGATAGTGCTGAGGGTGCTTTGAGAAATCAATACTTTAAGGACTATG
GCGAACGCTGGCACCCAGTAGCCAAAAAGAAAAATCAGACCATGATTGACATGG
TTATCAGCTTACTAGCTGAGGGACGCTTTTACTACCTTGACATTCCTGCTAACAA
GGTCTTTGTTGAGGAGCATAAGATGTACCGCTATGATGACAAATCTCTTAACTCT
GATGACCCCAAAGTTATCAAAGAAGATGACCATACGGTGGATGAGTTCAAGTAT
TTTGTCCTGGACAACGCTAGGGAGCTAGATTTGAAAGCCTAA

**GroupF2-phage_capsid_and_scaffold**
ATGTCATTTACAACACAGGCTTTACTTGATTTAGGGTTAACTGATGAGCAAGCAA
AAGAAGTATTTGCTTTGCGAGGTGCTGAGATTAAAGATAATCAAAGCGCACTTG
ATACATTAACTGCTGAACGGGATAGCCTAAAAACACAGTTGGAACATAATCAAG
CAGAAATGAAGAAGTTACAAGACGATGTTGAACTTAGCAAAGACTCAAAAGATG
CACTTGCTAAATTACAGTCAGAGTTTGATGATTTCAAAAAAACTGCTGATGAAAC
GTTGCAACAAACAATCAAGACAGATGCAATTAAGCTTGCAATTAAAGATACAAA
TGCACTTGATACAGATTTGATGATGAAATTGATTGATGTTGACACTGTTGAATTA
GATGACAATGGCAAACCTCAATTAGAAACAATTATCAATGAGTTGCAAGGAAGC
AAACCATTTTTATTTGCACCAGCTCAAGAACCATCTGAACAAGGTAATAGCAAAC
CAACTATCTTTAACAATGGCAACCCACCAGCAAACCCTGCTAAGACAGAGGTTG
ACCCGTTTGAGGCGGTTGTTAATAGCTATTTATAG

**GroupF2-terminase_large_subunit**
ATGCAAATAGTTAATATCCAAAAGAATATTAACCCTCACTTTAAAAGTGTTTGGT
TATCTAAAAAGCCTAACAACATCTTGCGAGGTGGTCGGAACTCTTTCAAATCGTC
TGTTATCACTCTAAAACTAATTTACATGATGATTAAGTACATCATTAGAGGTGAG
AGAGCTAACATAGTTGTCATTCGTAAGGTTGCTAACACATTAAGGGATAGTGTTT
ATAATCAAATACAATGGGGATTAAGGTTGTTTTGTATTATTGGCATGTTCAAAAT
GACAGTTAGTCCATTCAAGATAACTCATATCAAGACAGGATCAACATTTTATTTT
TATGGATTAGATGATTTCCAAAAACTGAAATCAAATAACATTGGTGATCTTATTG
CGGTATGGTATGAGGAGGCTGCTGAGTTTTCAAGTTATGAAGAATTTGACCAAAC
TAATATCACATTTATGCGTCAAAAGCATCCAAAAGCTGACTTTGTTCAATTCTTTT
GGTCATATAATCCACCTCGTAATCCTTACAATTGGATAAATGAGTGGTTTGAACA

ATGCAAGCAACATCCTGATTATCTATGTCACTCTAGCACTTATCTTGATGATGAG
TTGGGATTTGTTACACCGCAAATGTTAGCCGACATTGAACGTATTAAAGAGAATG
ATTATGACTATTACAGATATGTCTATCTTGGTGAGGCAGTCGGGTTAGGTAACAA
TGTATATAACATGAGTACCTTTCACGCGCTGGATGCATTGCCATCTGATGATAAG
CTTATTGGCATATCTTATGCACTCGATGGTGGACATCAGCAATCAGCAACCGCGG
TCTGTGCATTTGGTATCACGGCCAAAGGTAAAGTTATCTTGCTAGACACATGGTA
TTATTCACCAGCTGGCCAAGTGGTTAAGAAAGCACCAAGTCAGTTGACTCAAGA
GATTAATGCATTTATGCAGGGCATAACAGATAAGTACAAGGTGCAGACTTTACA
ATACACTATCGATAGTGCAGAGGGTGCTTTGCGAAATCAATTTTATTTGGACTTT
GCTATCAGATGGCATCCGGTGGCCAAGCTTAGAAAAGTAACCATGATTGACAAT
GCACAGTCGTTACTTGCTCAAGGTAGATTTTACTATCTTGATACAGAAAACAATA
AAGTATTTATTTCAGAGCATCGAATGTACAGGTGGGATGAAAAGACAATACACT
CAGACAATCCAAACGTTATCAAAGAAGATGATCATACATGCGATGTCTTTCAATA
TTTTGTTTTGGATAACTCAAGACTTTTGGGTCTTAGGGTTGGGAATAGTTAG

**Data S2.** Prophage-integrase database for the screening and classification of prophages by integrase type. The integrase sequences shown belong to the integrases identified in this study (GBS*Int*6.3 and GBS*Int*8.2), the sequences of the integrases identified by Crestani et al (Crestani et al., 2020) can be found at https://github.com/chcrestani/GBS_prophage_integrase_typing.

**GBS*Int*6.3**
MRIESYKKKNGTTAYRFRVYIGVIDGKKKYIKRSGFTSKKLAKQALINLQQEIENPKD
KSTLLFKDLTKIWLDNYEKTVQGSTYLKTKRNIENHILPSLGSYQIKDLTPLIIQKYAD
EWSTKLKYSSKIVGIVRNILNHAVKFQYITSNPSAPVSAPKIQRTINKKKDYYNKDEL
KEFMQLVYNTDDINIIATFRLLAFTGLRKGEMLALTWKDYRNGTLDVNKAITRDIAG
EHIGPTKNKSSDRLISLDPETMNVLDNLHKTYPKTKYILESASGRWISPTQPRRWLVQ
ILRDSISKLEPIRIHGFRHTHASLLFESGLTLKQVQHRLGHEDLKTTMNTYVHITETAK
DEIGTKFSKYIDF

**GBS*Int*8.2**
MWHEEQSNGKIKFIEYYKDPYTGKRKRAYVTLDRYTKQSENKARRMLNEIIDERIKS
SGDVYIRFGQLVDEWKLSHSKTVKARTMRVYKHPLEQIRAFIGDEVLVKNIDTRLLQ
KFVDGLKDKYADNTVNLIKQPLNMILDYAVRMDYIQINPMKNVITPKRKKITKKQLE
EKYLETEQNQKIIAELRDPVYGNHIANFAEVIFLTGMRPGELLALRWDHVDIDNLKIK
IEYTLDYSTNGHAKADIGTVKNDGSYRTIDMPLRVKEILIEEYNYQSLNDLKNDFIFIS
KNGNHLSINTINRRIKKTSKKLYGIVITSHSFRHGHITLLAELGIPLKSIMDRVGHTDV
NTTIKVYTHATDKIGKQMIDKINKFVPIQSL
